# Hyperfractionated Treatment with ^177^Lu-Octreotate Increases Tumor Response in Human Small-Intestine Neuroendocrine GOT1 Tumor Model

**DOI:** 10.3390/cancers14010235

**Published:** 2022-01-04

**Authors:** Mikael Elvborn, Emman Shubbar, Eva Forssell-Aronsson

**Affiliations:** 1 Department of Medical Radiation Sciences, Institute of Clinical Sciences, Sahlgrenska Academy, University of Gothenburg, SE-413 45 Gothenburg, Sweden; emman.shubbar@gu.se (E.S.); eva.forssell_aronsson@radfys.gu.se (E.F.-A.); 2 Sahlgrenska Center for Cancer Research, Sahlgrenska Academy, University of Gothenburg, SE-413 45 Gothenburg, Sweden; 3 Department of Medical Physics and Biomedical Engineering, Sahlgrenska University Hospital, SE-413 45 Gothenburg, Sweden

**Keywords:** GEP-NET, midgut carcinoid, DOTATATE, BALB/c, somatostatin, radiopharmaceutical, radionuclide therapy, PRRT, Lutathera

## Abstract

**Simple Summary:**

Neuroendocrine tumors are slow growing and initially associated with vague symptoms and, therefore, often spread in the patient’s body at diagnosis, leading to a poor prognosis without means of curation through surgery. Although tumor-targeting treatments exist and are used in clinics, they are not fully optimized. The aim of this study was to test different dosages and time intervals of the radioactive pharmaceutical ^177^Lu-octreotate. We found that dividing a dosage into several portions and administering it at short time intervals resulted in a stronger tumor reduction and/or prolonged time for regrowth in mice than if given as a single dose. The biggest differences were seen in the lower dosage levels of the study. The findings indicate that there is clear room for improvements in the treatment of neuroendocrine tumors with ^177^Lu-octreotate.

**Abstract:**

Radionuclide treatment of patients with neuroendocrine tumors has advanced in the last decades with favorable results using ^177^Lu-octreotate. However, the gap between the high cure rate in animal studies vs. patient studies indicates a potential to increase the curation of patients. The aim of this study was to investigate the tumor response for different fractionation schemes with ^177^Lu-octreotate. BALB/c mice bearing a human small-intestine neuroendocrine GOT1 tumor were either mock treated with saline or injected intravenously with a total of 30–120 MBq of ^177^Lu-octreotate: 1 × 30, 2 × 15, 1 × 60, 2 × 30, 1 × 120, 2 × 60, or 3 × 40 MBq. The tumor volume was measured twice per week until the end of the experiment. The mean tumor volume for mice that received 2 × 15 = 30 and 1 × 30 MBq ^177^Lu-octreotate was reduced by 61% and 52%, respectively. The mean tumor volume was reduced by 91% and 44% for mice that received 2 × 30 = 60 and 1 × 60 MBq ^177^Lu-octreotate, respectively. After 120 MBq ^177^Lu-octreotate, given as 1–3 fractions, the mean tumor volume was reduced by 91–97%. Multiple fractions resulted in delayed regrowth and prolonged overall survival by 20–25% for the 120 MBq groups and by 45% for lower total activities, relative to one fraction. The results indicate that fractionation and hyperfractionation of ^177^Lu-octreotate are beneficial for tumor reduction and prolongs the time to regrowth.

## 1. Introduction

Many neuroendocrine tumors (NETs) are characterized by the overexpression of somatostatin (SS) receptors (SSTRs) on their cell membrane, allowing treatment with peptide receptor radionuclide therapy (PRRT). Today, ^177^Lu-[DOTA0,Tyr3]-octreotate (^177^Lu-octreotate, ^177^Lu-DOTATATE, Lutathera^®^) is approved by the FDA and EMA for treatment of patients with somatostatin-receptor-positive gastroenteropancreatic (GEP) NETs, unresectable or metastatic, and progressive and well differentiated (grades 1–2), with a regimen of 4 administrations of 7.4 GBq 7–9 weeks apart [1].

The most beneficial clinical effects today for ^177^Lu-octreotate treatment, administered in up to 4 cycles, are prolonged survival and improved quality of life; a few percent of patients reported complete remission (CR), up to 30% partial remission (PR), and 35% stable disease (SD) [2,3]. The main organs at risk in treatment using ^177^Lu-octreotate are the bone marrow and kidneys, with transient deprivation of blood cells as acute effects, and delayed effects as loss of renal function and blood malignancies, e.g., leukemia and myelodysplastic syndrome [3,4]. To handle the acute effects on bone marrow, the treatment is given as several administrations (cycles) with enough time between cycles for bone marrow recovery, 7–9 weeks [1]. To avoid late nephrotoxicity, the number of cycles is limited and the absorbed dose limit suggested for kidneys is usually 23 or 28 Gy [5,6,7]. The biokinetics of ^177^Lu-octreotate varies between patients, and the absorbed dose to the kidneys per administered activity varies up to a factor of 8 between patients [8]. Thus, most patients could receive more than 4 cycles [9], and the majority of patients are probably undertreated with the current treatment schedule.

Compared with the results in animal NET-models, the effects of patient NET treatments are still modest. In animal studies with SSTR_2_-expressing tumors, CR in up to 96% and even 100% of the individuals have been achieved from single injections with ^177^Lu-octreotate [10,11,12,13,14]. The difference in treatment response testifies to the existence of major possibilities for improvement in the therapy of patients with NETs [15].

It has previously been demonstrated in mice that the biodistribution and dosimetry of radiolabeled SS analogues are strongly influenced by the amount of injected peptide [16,17]. At high amounts of peptides, the reduced binding and uptake can be explained by SSTR saturation, i.e., if the number of receptors expressed by the tumor cells is limited and lower than the number of available receptor-binding peptides in the tumor, no further binding can occur when all receptors are occupied. Binding of SS analogues to some of the SSTR subtypes, e.g., octreotide to SSTR_2_, will result in internalization of the complex via receptor-mediated endocytosis, followed by a subsequent recycling of receptors to the cell membrane [18,19,20,21,22]. The implications of SSTR saturation have also been studied and discussed in animal models and patient studies previously [17,23,24,25,26,27]. It is therefore reasonable to assume that the binding, retention, and absorbed dose to the tumor tissue would increase if a single cycle of ^177^Lu-octreotate treatment was divided into smaller fractions with a time interval between them, allowing the SSTRs to recycle and, thereby, lower the effects of saturation.

Most studies of biodistribution and therapy of radiolabeled SS analogues on NET-bearing mice have used a single administration of various amounts. There are, however, both in vitro and in vivo studies demonstrating radiation-induced upregulation of SSTRs [28,29,30,31]. In addition, there are studies demonstrating higher binding and therapeutic effects after administering a small amount of ^177^Lu-octreotide 24 h prior to the main treatment, sometimes referred to as ‘priming’ [32,33]. In those studies, the first small amount was used to increase SSTR expression. More prominent treatment responses have also been achieved using a dual-fractionation scheme with a reduced amount of radioactivity [13]. A patient case study reported higher uptake and prolonged retention of ^111^In in the tumor tissue, and almost doubled the estimated absorbed dose to the tumor, when splitting one cycle of ^111^In-DTPA-D-Phe^1^-octreotide into two smaller fractions administered 24 h apart [27].

Although there have been some studies about fractionated radionuclide therapy, the knowledge gaps in this area are large. Optimization of dosing and timing between the cycles/fractions in ^177^Lu-octreotate treatment could increase the absorbed dose and therapeutic effects on tumors.

The aim of this study was to investigate the effects of fractionated and hyperfractionated ^177^Lu-octreotate administration on human small-intestine neuroendocrine GOT1 tumors in mice, using varying amounts of ^177^Lu-octreotate and time intervals between administrations.

## 2. Materials and Methods

### 2.1. Radiopharmaceutical

^177^Lu was obtained as ^177^LuCl_3_ and bound to DOTA^0^-Tyr^3^-octreotate according to the manufacturer’s instruction (I.D.B. Holland, Baarle-Nassau, Netherlands). The peptide-bound fraction was determined by instant thin-layer chromatography, ITLC-SG (Varian, Lake Forest, CA, USA), using 0.1 mL/L sodium citrate (pH = 5; LabService AB, Sundsvall, Sweden) and it was >93% at all administrations. Saline solution was used to dilute the stock solution to the desired activity concentrations. Syringes were filled with ^177^Lu-octreotate and measured in a well-type ionization chamber (CRC-15R; Capintec, Ramsey, NJ, USA) before and after administration, to determine the administered amount of ^177^Lu-octreotate to each animal.

### 2.2. Animal Experiments

Forty-three female BALB/c-mice were transplanted s.c., at the age of 5–6 weeks, with small pieces of tumors (ca 1 mm^3^) from the patient-derived small-intestine NET GOT1 [34]. The tumor pieces used for transplantation came from serial transplanted tumors that originated from mice inoculated with GOT1 cells a few generations ago. Transplanted tumor pieces grew until the start of treatment. At the start of treatment, mice (aged 26 ± 9 weeks (SD), median 25 weeks) were divided into groups of 3–5, as evenly as possible with respect to tumor size and age, and then intravenously injected in one tail vein with ^177^Lu-octreotate or saline. Data on the groups and administration schedules are presented in Table 1. The mice had access to standard laboratory food and water ad libitum and were kept under a standard laboratory day and night cycle, i.e., dark from 6 pm to 6 am. The experiments were approved by the Ethical Committee for Animal Research at the University of Gothenburg (No. 107-2015).

Tumor sizes were generally measured with calipers, twice weekly after the start of treatment, until the mice met any of the criteria: tumor weight >10% of body weight, >10% loss of body weight, or general poor health condition. From these data, overall survival (OS) and progression-free survival (PFS) were determined. Tumor progression was defined as the time, after the initial treatment response (if any), when a tumor was larger than at the start of treatment. The mice were killed by cardiac puncture under anesthesia with sodium pentobarbital (APL, Kungens Kurva, Sweden), and the remaining tumor tissues were collected and divided, and parts were fixed in formalin or snap frozen in liquid nitrogen, and stored at −80 °C.

### 2.3. Immunohistochemical Analyses of SSTR_2_ and CgA (IHC)

The resected tumor tissues, formalin-fixed and paraffin-embedded in tissue blocks (FFPE), were sectioned in 4 μm slices and put on FLEX IHC microscope slides (Dako, Stockholm, Sweden). The sections were then deparaffinized, rehydrated, and processed with Dako EnVision^TM^ FLEX antigen retrieval EDTA buffer pH 9 using the DAKO PT Link module (PT Link, DakoCytomation, Glostrup, Denmark) according to the manufacturer’s instructions. The staining was performed in an autostainer (DAKO Autotstainer Plus, DakoCytomation, Glostrup, Denmark) according to manufacturer’s instructions. A human small intestinal NET was used as a positive control for somatostatin receptor 2 (SSTR_2_) and chromogranin A (CgA). Applied antibodies were rabbit anti-SSTR_2_ (ab134152, 1:50; Abcam, Cambridge, UK) and rabbit anti-CgA (ab68271, 1:500; Abcam, Cambridge, UK). The stained tissue sections were reviewed and assessed by two certified pathologists. Images for the article were digitalized using a Leica SCN400 and SlidePath Gateway Client LAN 2b4 (Leica Microsystems, Buffalo Grove, IL, USA).

### 2.4. Data Analysis

Tumor volume, relative to volume at the start of treatment, was plotted versus the corresponding time point. The area under the curve, AUC, was calculated using the trapezoid rule for each individual mouse. To obtain matching measurement points on the few occasions where there were seven days between measurements, linear interpolation was performed between the closest measurements to estimate the missing value. The AUC for each significance test for a group was calculated from the start of treatment until the day the first mouse was killed in the group.

The test for determination of statistical significance was performed in IBM SPSS Statistics 26 (IBM Svenska AB, Stockholm, Sweden). For the three groups that received a total amount of 120 MBq of ^177^Lu-octreotate, a Kruskal-Wallis one-way analysis of variance was conducted. For the groups that received a total amount of 30 or 60 MBq of ^177^Lu-octreotate, a pairwise Mann-Whitney *U* test was used. Statistical significance was considered for probabilities above 95% (*p* < 0.05).

## 3. Results

### 3.1. Effects of Low-Medium Total Amounts of ^177^Lu-Octreotate (30–60 MBq)

**Figure 1:**
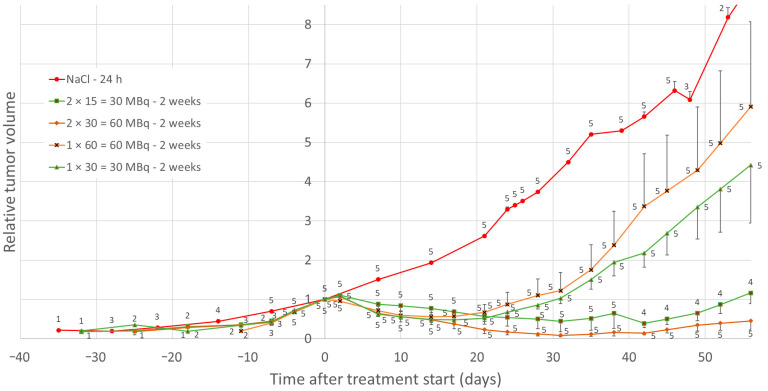
Relative tumor volume for mice that received a total activity of 30 (green triangles and rectangles) or 60 (brown diamonds and crosses) MBq ^177^Lu-octreotate. Mock-treated mice (red dots) received saline 24 h apart, with time offset to match tumor size at the start of treatment. The detailed treatment schemes for the groups are shown in Table 1. Data are given as mean values and error bars indicate standard error of the mean (SEM). Data labels indicate group size at the given time point.

Maximal tumor volume reduction for groups was seen after 2 weeks for single administrations of ^177^Lu-octreotate, when the mean tumor volume was reduced by 52% and 44% for groups 1 × 30 and 1 × 60 MBq, respectively. For double administrations, the maximal mean tumor reduction was 61% after 6 weeks and 91% after 4 weeks for 2 × 15 = 30 and 2 × 30 = 60 MBq groups, respectively (Figure 1).

A statistically significant difference in AUC for relative tumor volume, calculated individually for each mouse, was found between the groups that received 2 × 30 and 1 × 60 MBq of ^177^Lu-octreotate until day 56 after the start of treatment (*p* = 0.008). No statistically significant difference in AUC was found between the groups that received 2 × 15 and 1 × 30 MBq of ^177^Lu-octreotate until day 38. One mouse in the 2 × 15 MBq group did not respond, and when that mouse was excluded, there had been a statistically significant difference until day 59 (*p* < 0.02).

**Figure 2:**
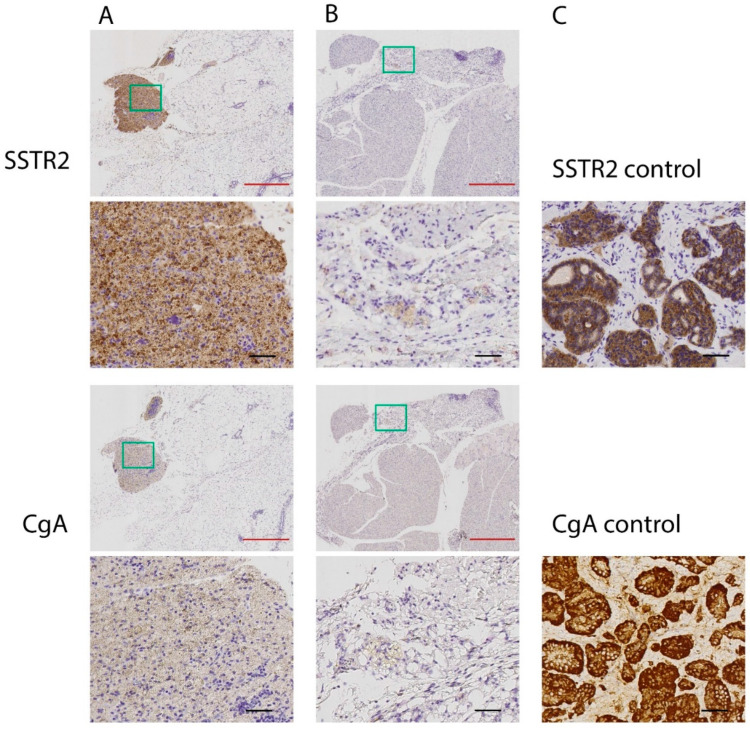
Immunohistochemical analyses of the expression of somatostatin receptor 2 (SSTR_2_) and chromogranin A (CgA) in the residual tissue from the two mice whose tumors receded completely (panel (**A**,**B**) respectively) after treatment with 2 × 30 = 60 MBq of ^177^Lu-octreotate. Results are presented in two magnifications, where the green box in the upper image demonstrates the location of the magnified image below, for each sample and staining. Two certified pathologists reviewed and assessed the stained sections. Weak staining was found for both SSTR_2_ and CgA in most parts of the residual tumor tissue samples (**A**,**B**), except for a small highly stained part in panel (**A**). Evaluation of the tissues concluded that no or very few malignant tumor cells were present in the sections, and that positive staining was mainly related to lymphocytes. Positive controls are demonstrated in panel (**C**), where a human small intestinal NET was used as a positive control for SSTR_2_ and CgA expression. Black bar = 50 µm, red bar = 500 µm.

**Figure 3:**
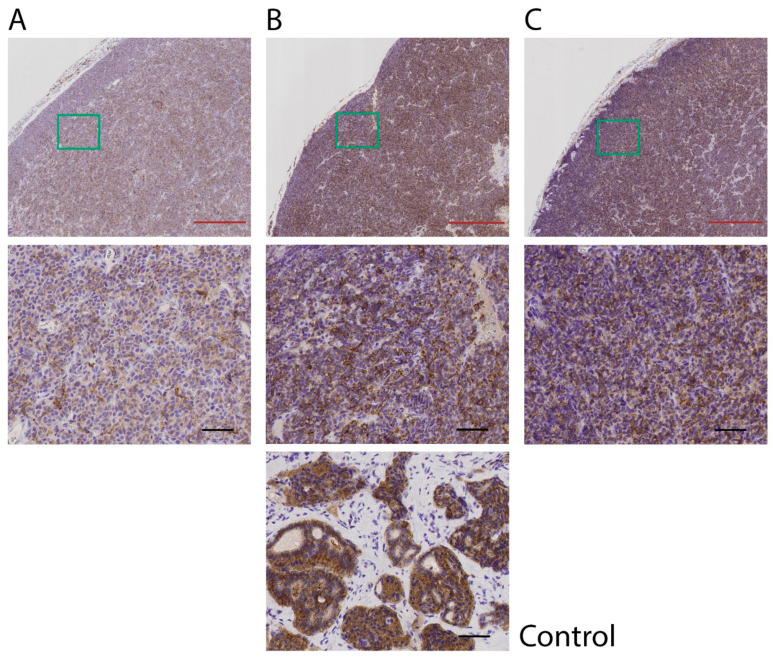
SSTR_2_ expression in three regrown GOT1 tumors from mice treated with 2 × 15 = 30 MBq of ^177^Lu-octreotate, analyzed immunohistochemically. Results are presented in two magnifications, where the green box in the upper image demonstrates the location of the magnified image below, for each sample. SSTR_2_-staining was positive in all tumor sections, although the mouse without tumor reduction (**A**) expressed lower expression of SSTR_2_ than the other two mice (**B**,**C**). Human small intestinal NET was used as a positive control. Two certified pathologists reviewed and assessed the stained sections. Black bar = 50 µm, red bar = 500 µm.

For two mice in the 2 × 30 = 60 MBq-group, the tumors completely regressed, leaving residual tissues of about the size of a pinhead. Histopathological analysis of these residual tissues showed overall weak positive CgA and SSTR_2_ staining, except for a small highly stained part in one of the tumors with higher CgA and SSTR_2_ staining (Figure 2). In the remaining parts of the sections, no or few malignant tumor cells were present, and positive staining was, to a high degree, related to lymphocytes. The highly stained part in tumor A mainly consisted of degraded cell material with very few cells, mainly lymphocytes and fibroblasts. If there were any remaining tumor cells, the amount was very low. The staining was highest in the degraded cell material. For one mouse in the 2 × 15 = 30 MBq group, tumor volume reduction did not occur after treatment, and the expression of SSTR_2_ in this tumor tissue was lower than for the other tumor tissues in this group (Figure 3).

**Figure 4:**
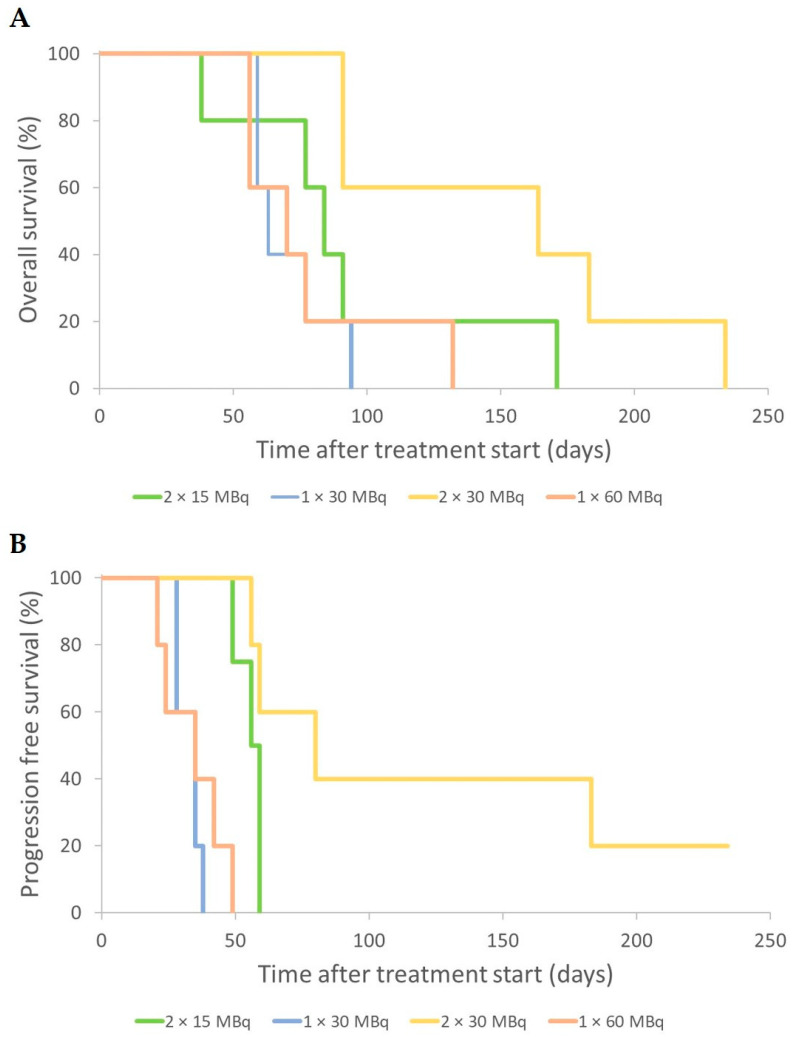
Overall survival (**A**) and progression-free survival (**B**) for mice that received an activity of 2 × 15 MBq (green), 1 × 30 MBq (blue), 2 × 30 MBq (yellow), and 1 × 60 MBq (brown) of ^177^Lu-octreotate. Tumor progression was defined as the time, after initial treatment response (if any), when a tumor was larger than at the start of treatment, or when an animal was killed.

The median OS was 84 days (range 38–171 days) for the 2 × 15 = 30 MBq group, 63 days (59–94 days) for 1 × 30 MBq, 164 days (91–234 days) for 2 × 30 = 60 MBq, and 70 days (56–132 days) for 1 × 60 MBq group (Figure 4A). Median PFS was 56 days (range 49–59 days) for 2 × 15 = 30 MBq, 35 days (28–38 days) for 1 × 30 MBq, 80 days (56–>234 days) for 2 × 30 = 60 MBq, and 35 days (21–49 days) for 1 × 60 MBq group (Figure 4B).

### 3.2. Effect of High Total Amounts of ^177^Lu-Octreotate (120 MBq)

**Figure 5:**
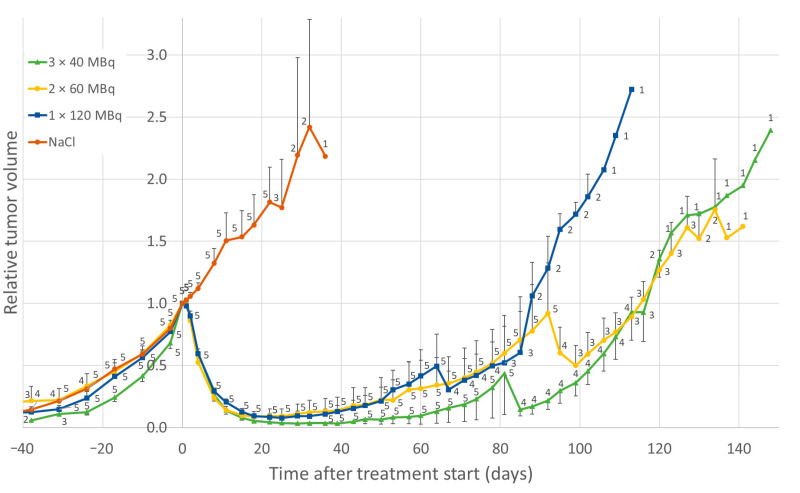
Relative tumor volume in GOT1-bearing mice before and after treatment with ^177^Lu-octreotate, normalized to the tumor volume at the start of treatment (day 0). Green triangles indicate an activity of 3 × 40 MBq, yellow circles 2 × 60 MBq, blue squares 1 × 120 MBq of ^177^Lu-octreotate, and brown circles saline only, with multiple fractions given 24 h apart. Detailed description of the treatment schedules of the groups is given in Table 1. Data are given as mean values, and error bars indicate standard error of the mean (SEM). Data labels indicate group size at the given time point.

The group of mice that received a single administration of 120 MBq ^177^Lu-octreotate displayed maximal tumor volume reduction after 3 weeks, when the mean tumor volume was reduced by 92%. Double and triple administrations of ^177^Lu-octreotate (at 24 h apart, and a total of 120 MBq administered) resulted in 91% and 97% tumor volume reduction at weeks 3–4, respectively (Figure 5). No statistically significant differences were found in the AUC for tumor volume between the three groups that received a total of 120 MBq of ^177^Lu-octreotate, during the first 64 days.

**Figure 6:**
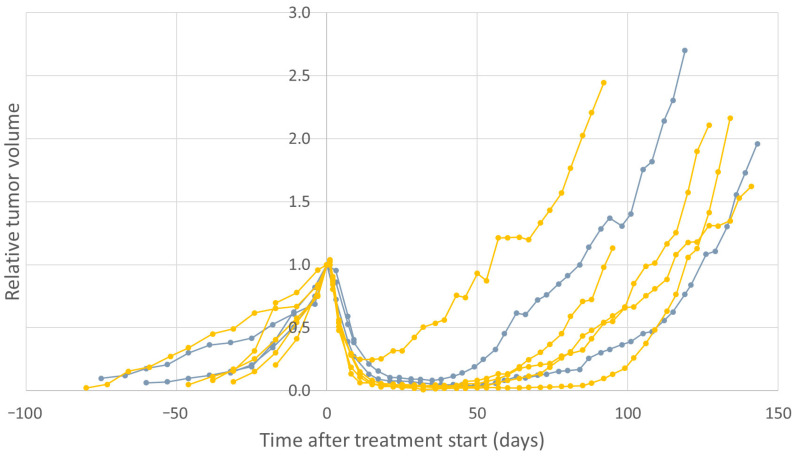
Relative tumor volume in GOT1-bearing mice that received daily (yellow, *n* = 5) or weekly (gray, *n* = 3) injections of an activity of 2 × 60 MBq to a total of 120 MBq of ^177^Lu-octreotate. One mouse in the weekly treated group lost body weight and was euthanized nine days after the start of treatment.

Among the mice that were treated with 2 × 60 MBq ^177^Lu-octreotate, there was no visible difference in the regrowth response between the ones that received daily administrations and the ones that received weekly administrations (Figure 6). One mouse in the “once weekly-treated group” lost body weight and was euthanized nine days after the start of treatment.

**Figure 7:**
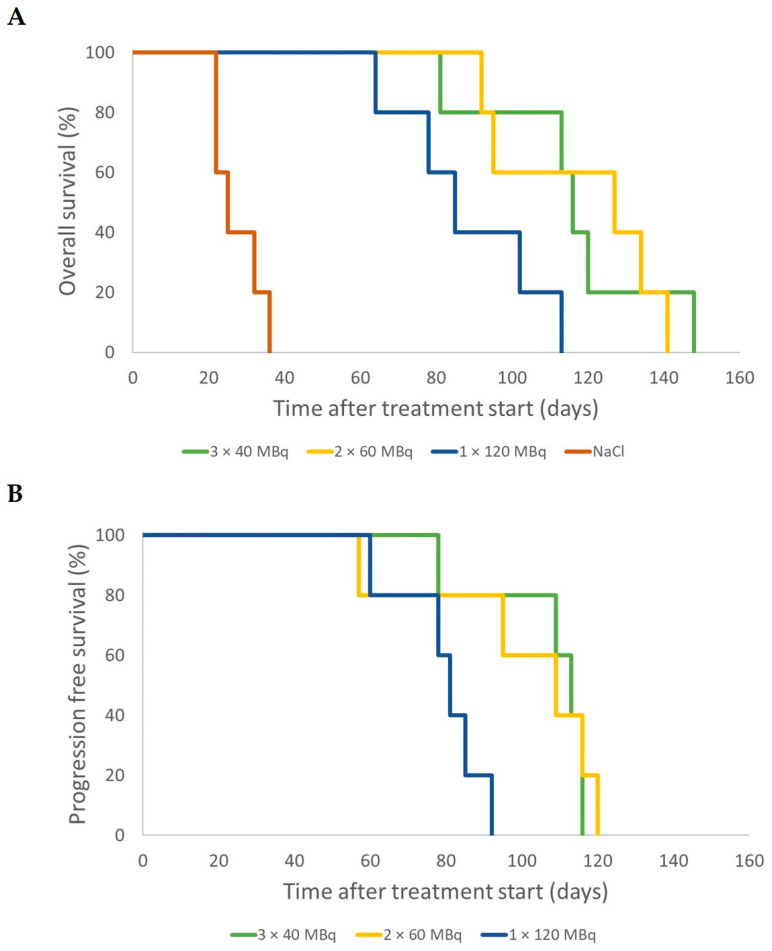
Overall survival (**A**) and progression-free survival (**B**) for mice that received an activity of 3 × 40 MBq (green), 2 × 60 MBq (yellow), 1 × 120 MBq (blue) of ^177^Lu-octreotate, or saline (NaCl) only (brown). Tumor progression was defined as the time, after initial treatment response (if any), when the tumor was larger than at the start of treatment, or when the animal had to be killed. Detailed description of the treatment schedules of the groups is given in Table 1.

The median OS was 25 days (range 22–36 days) for the control group, 116 days (81–148 days) for 3 × 40 MBq, 127 days (92–141 days) for 2 × 60 MBq, and 85 days (64–113 days) for 1 × 120 MBq. The maximal overall survival after 2–3 administrations was prolonged by 20–25% compared to a single dose (Figure 7A). The median PFS was 113 days (78–116 days) for 3 × 40 MBq, 109 days (57–120 days) for 2 × 60 MBq, and 81 days (60–92 days) for 1 × 120 MBq (Figure 7B).

## 4. Discussion

The present study demonstrated a distinct tumor volume reduction for the groups that received more than one administration with the same total amount of ^177^Lu-octreotate, compared with a single administration. Similar findings after fractionation with two administrations were presented from previous studies in the same model [32,33], and in a previous study on SSTR-expressing human small-cell lung cancer in mice, although this issue was a minor part of the study [13]. Higher therapeutic effects were also reported in mice with rat exocrine pancreatic tumor AR42J after fractionation of ^177^Lu-octreotate or ^67^Cu-sartate, given 2 weeks apart [35]. Furthermore, in a patient with small intestine NET, fractionation (two administrations 24 h apart) led to a higher tumor uptake of ^111^In-octreotide compared to a single injection [27].

Two explanations for higher tumor binding and uptake and effect on tumor growth after fractionation and hyperfractionation are (1) reduction of saturation of SSTRs when therapeutic amounts of ^177^Lu-octreotate are used, and (2) radiation-induced upregulation of SSTR expression from the first administration.

Saturation of SSTRs has been shown in studies on binding and internalization of ^111^In-octreotide and ^177^Lu-ocetrotate to various NET cells in primary culture, demonstrated as relatively higher binding after incubation with lower concentrations of the radiolabeled SS analogue [20]. SSTR saturation has also been shown in GOT1 and other NET animal models using radiolabeled SS analogues [17,25,36]. In SSTR-expressing normal tissues in rats, a bell-shaped binding curve for ^111^In-octreotide was reported [24]. In a similar study using the GOT1 mouse model, SSTR saturation was found both in GOT1 tumors and in SSTR-expressing normal tissues, and in some cases, there was a trend towards a bell-shaped binding curve vs. injected amount of ^111^In-octreotide [17]. If fractionated treatment is given with enough time between administrations, SSTR recycling can occur and SSTR expression can be restored on the cell membrane. The time for SSTR internalization and recycling is, to our knowledge, not known in detail, and various values are discussed. In a study on rats transplanted with AR42J tumors, internalization of SSTR_2_ after administration of SS agonists was fast (already detected after 2.5 min) with restored receptors at the cell membrane after 6–24 h [21].

Radiation-induced upregulation of SSTRs was shown in vitro in human small-cell lung cancer [30,31] and in vivo in the GOT1 animal model [28,29]. The in vitro studies clearly demonstrated higher binding of ^177^Lu-octreotate and higher mRNA expression of SSTR subtypes no. 1, 2, and 5, 1–7 days after external irradiation by low-to-high absorbed doses [30,31]. The first in vivo study in the GOT1 animal model showed that curative amounts of ^177^Lu-octreotate resulted in lower expression of SSTRs during the time period studied: 4 h to 13 days (reflected by lower tumor uptake of subsequently injected ^111^In-octreotide) [28]. However, non-curative amounts of ^177^Lu-octreotate also gave reduced SSTR expression initially at 4 h and 24 h, but at 3–13 days, the SSTR expression increased and resulted in about two times higher activity concentration in the tumor with the second administration than in the untreated mice. Since the early effect at 4 h was similar irrespective of the activity amount administered, the mechanisms behind the finding cannot be explained by rapid internalization or saturation of SSTRs, and are still unknown to us. The second in vivo study in the same mouse model was focused on effects after administration of non-curative amounts of ^177^Lu-octreotate, and confirmed the results from the first study [29]. Thus, radiation-induced upregulation of SSTR started after a few days and persisted for at least 1.5 weeks. Furthermore, the last study also demonstrated that the SSTR upregulation only occurred in the tumor tissue and not in normal tissues, not even in those with a normally high SSTR expression (e.g., adrenals and pancreas). Similar findings have also been presented for another animal model [37]. Furthermore, the dedicated studies on the ‘priming’ schedule further support radiation-induced SSTR upregulation in the GOT1 mouse model [32,33]. In summary, administration of 5 MBq 24 h before administration of 10 MBq resulted in ca 2 times higher absorbed dose to the tumor than after a single administration of 15 MBq [32]. The study also showed similar or even lower absorbed doses to the major normal organs, e.g., the kidneys. In addition, at this lower activity level, statistically significant better therapeutic effects were obtained, supporting the findings in the present work. Furthermore, transcriptomic analyses of GOT1 tumor tissues demonstrated higher proapoptotic and antiproliferative responses after the ‘priming schedule’, as well as increased stress response and initiation of cell cycle arrest [32,33].

The largest differences in treatment response were found in the low–medium dose groups, where the multiple fractions distinguished themselves. The decrease in maximal mean tumor volume for the 2 × 15 = 30 MBq group, although similar to the 1 × 30 MBq group, provided a substantially longer time to regrowth. The maximal tumor reduction in the 2 × 15 = 30 MBq group would be 82% at week 4 without the deviant mouse in this study, and the recession curve would look more like the one for the 2 × 30 = 60 MBq group. Interestingly, the two single-dose groups in this study (1 × 30 and 1 × 60 MBq) responded in a similar fashion in terms of tumor regression and regrowth patterns (*p* > 0.05), where the tumor regrowth appeared faster (and at about the same time after treatment) than for the groups that received multiple administrations. SSTR saturation in the tumors at treatment is most probably the cause of the similarity of these single-dose groups in terms of treatment response, which is in line with our previous therapeutic findings from a single administration of ^177^Lu-octreotate and with biokinetic data on ^111^In-octrotide [11,17]. The two groups with corresponding amounts of ^177^Lu-octreotate given as two administrations (2 × 15 and 2 × 30 MBq) also responded in a fairly similar way, although the group that received a higher total amount resulted in increased tumor reduction and longer time to regrowth. The latter group (2 × 30 MBq) was the only one in which the tumor regressed completely for two of the mice. The effects found in the four low–medium activity groups indicate that the number of fractions is key, as well as the amount of ^177^Lu-octreotate in each fraction, rather than the total amount of ^177^Lu-octreotate received. Since the treatment schedule for these studies using lower ^177^Lu activities included fractionation with a rather long time between fractions, the enhanced effects might be interpreted as being due to radiation-induced increased SSTR expression.

One of the mice in the 2 × 15 MBq group unexpectedly did not respond to ^177^Lu-octreotate treatment. It is known from the clinic that all patients do not respond to ^177^Lu-octreotate therapy and tumor progression occurs during treatment in 10–20% of the patients [2,9,38]. A probable explanation for the non-responding mouse in the present study could be a difference in SSTR expression in the tumor in that individual mouse, a fact that was also demonstrated by the immunohistochemical analyses. The GOT1 tumor model is unique, since it is probably the only small-intestinal NET cell line that could be transplanted to nude mice with preserved neuroendocrine features and receptor expression [34,39,40]. Since GOT1 cell proliferation is slow, most GOT1 animal studies are performed in the model based on serial transplantation of small tissue pieces from one tumor to several younger mice, which was the case in the present study. Due to some heterogeneity in GOT1 tumors [32,41], there is a risk that a mouse may be transplanted with a tumor piece having cells with altered SSTR expression, which was most probably the case in the present study, and explains the lower SSTR_2_ expression in this non-responding mouse. We reported similar findings in a previous study [32].

For the high-dose groups (120 MBq), there was a slight difference in tumor volume reduction between the groups, although not statistically significant, probably due to the high therapeutic effect altogether, with a reduced opportunity to statistically demonstrate any differences between groups. There was, however, a clear difference in tumor regrowth for the groups that received a fractionated schedule, which could be explained by more cells being killed with multiple fractions, leading to prolonged time to regrowth. The schedule with 24 h between fractions (hyperfractionation) will most probably be related to lower effects of SSTR saturation (see above). Furthermore, these results imply that 24 h between fractions is enough for recycling of SSTRs in vivo.

Altogether, the interpretation of the results in the present study is that higher tumor volume reduction and better prognosis are achieved if the total amount of ^177^Lu-octreotate is administered in fractions. The time between two fractions would give the SSTRs time to recycle to the cell membrane. This mechanism would result in higher internalization of the radiopharmaceutical in total and higher absorbed dose to the tumor, which would result in more damage to the tumor cells. However, it would be imperative to further investigate the main organs at risk, i.e., bone marrow and kidneys, to make sure that their radiation toxicity would not increase to the same extent. As discussed above, for the kidneys, our previous findings from comparing administration of one or two fractions, showed an even lower absorbed dose to the kidneys after fractionation [32], indicating an advantage regarding nephrotoxicity. However, further studies on different hyperfractionation protocols should be performed, especially focused on kidneys and bone marrow to avoid a potential higher risk of side effects [3,4]. Since the shelf life of the product Lutathera^®^ is 72 h from the time of calibration [1], it would be feasible to treat a patient up to three consecutive days with the content of one vial of ^177^Lu-octreotate.

## 5. Conclusions

The results of the present study indicate that a fractionated or hyperfractionation schedule with ^177^Lu-octreotate would be beneficial in order to increase the therapeutic effects on neuroendocrine tumors. Therefore, more studies on the effects and mechanisms behind tumor response and the effects on organs at risk with hyperfractionation is planned. Hyperfractionation of ^177^Lu-octreotate may be one option to consider to advance the health care for patients with neuroendocrine tumors in the future.

## Figures and Tables

**Table 1:** Overview of the studies performed. A total amount of 30, 60, or 120 MBq ^177^Lu-octreotate was administered, given as one, two, or three fractions, with the indicated time between administrations. One group received physiological saline (NaCl) only, as a mock treatment. Time between injections were 24 h, 7 days (1 week), or 14 days (2 weeks). The tumor volume was given at the start of treatment, and presented as mean ± standard error of the mean (SEM).

Total Activity	Activity			Time Apart	Tumor Volume [mm^3^]	Group Size
	1st Injection	2nd Injection	3rd Injection			
30 MBq	15 MBq	15 MBq	-	2 weeks	520 ± 70	*n* = 5
30 MBq	30 MBq	NaCl	-	2 weeks	530 ± 50	*n* = 5
60 MBq	30 MBq	30 MBq	-	2 weeks	400 ± 30	*n* = 5
60 MBq	60 MBq	NaCl	-	2 weeks	350 ± 30	*n* = 5
120 MBq	60 MBq	60 MBq	NaCl	1 week	1100 ± 40	*n* = 3
120 MBq	40 MBq	40 MBq	40 MBq	24 h	1400 ± 90	*n* = 5
120 MBq	60 MBq	60 MBq	NaCl	24 h	1300 ± 80	*n* = 5
120 MBq	120 MBq	NaCl	NaCl	24 h	1600 ± 90	*n* = 5
Mock treatment	NaCl	NaCl	NaCl	24 h	1300 ± 90	*n* = 5

## Data Availability

Data are contained within the article.
